# NEDD1 overexpression increases cell proliferation, tumor immune escape, and drug resistance in LUAD

**DOI:** 10.7150/jca.91671

**Published:** 2024-03-11

**Authors:** Ting Zhuo, Zuotao Wu, Sirong Chen, Chuyi Yang, Hongyu Huang, Jinyan Gan, Jueqi Lyu, Juan Xiao, Zihao Li, Shouming Qin, Yanbin Wu

**Affiliations:** 1 Department of Pulmonary and Critical Care Medicine, The First Affiliated Hospital of Guangxi Medical University, Nanning 530021, China.; 2 Department of Cardio-Thoracic Surgery, The First Affiliated Hospital of Guangxi Medical University, Nanning 530021, China.; 3 Department of Radiotherapy, Guangxi Medical University Cancer Hospital, No. 71 Hedi Rd, Nanning, Guangxi Zhuang Autonomous Region, 530021, China.; 4 Department of Pediatrics, The First Affiliated Hospital of Guangxi Medical University, Nanning 530021, China.

**Keywords:** NEDD1, LUAD, cell proliferation, immune infiltration, drug resistance

## Abstract

**Background:** Neural Precursor Cell Expressed Developmentally Down-Regulated Protein 1 (NEDD1) serves as a crucial factor in promoting cellular mitosis by directly facilitating wheel assembly and daughter centriole biogenesis at the lateral site of parent centrioles, ultimately driving centrosome replication. The amplification of centrosomes and the abnormal expression of centrosome-associated proteins contribute to the invasion and metastasis of non-small cell lung cancer cells. However, the specific mechanism by which NEDD1 contributes to the progression of lung adenocarcinoma (LUAD) remains unexplored. Therefore, the objective of this study is to uncover the role played by NEDD1 in LUAD.

**Methods:** To verify the expression of NEDD1 in pan-carcinoma. The feasibility of NEDD1 as a prognostic marker for LUAD in TCGA and GEO databases was verified. Subsequently, Cox proportional hazard regression analysis was used to screen the prognostic factors of LUAD, so as to analyze the correlation between prognostic factors and NEDD1 expression. For another, NEDD1-related genes were screened for pathway enrichment analysis to verify their possible functions. In addition, the expression of NEDD1 in LUAD was verified by qPCR and IHC, then siRNA was used to construct NEDD1-knocked lung cancer cells for subsequent cytobehavioral experiments. Finally, the distribution of NEDD1 in single-cell samples was revealed, and then the correlation between its overexpression and LUAD immune escape and drug resistance was analyzed.

**Results:** LUAD exhibits upregulation of NEDD1, which in turn promotes the proliferation, migration, invasion, and epithelial-mesenchymal transition of lung cancer cells, thereby contributing to a poor prognosis. Furthermore, the overexpression of NEDD1 is closely associated with immune escape and drug resistance in LUAD.

**Conclusion:** NEDD1 serves as a reliable prognostic marker for LUAD, and its upregulation is associated with increased immune escape and drug resistance. Given these findings, NEDD1 holds potential as a novel therapeutic target for the treatment of LUAD.

## Introduction

The microtubule cytoskeleton plays a crucial role in facilitating directed and orderly molecular and organelle movements within cells. This network is particularly essential for ensuring the accurate separation of chromosomes during cell division. 1 The microtubule network can drive microtubule nucleation by distributed on centrosomes, around chromosomes, and pre-existing microtubules, and when the cell enters the late stage of division, the spindle pulls all organelles arranged on the equatorial plate for normal cell division. The realization of these pathways requires γ-tubulin loop complex (γ-TuRC) and its adaptor protein NEDD1. 2 Furthermore, NEDD1 plays a direct role in activating the assembly of the wheel and the biogenesis of subcentrioles in the lateral position of the parent centrioles, thereby promoting centrosome replication. 3, 4

As the most prevalent form of lung cancer, LUAD (lung adenocarcinoma) is associated with a high mortality rate and is characterized by its highly heterogeneous nature. While surgical resection can cure LUAD at early stages, there remains a significant risk of recurrence. 5, 6 Extensive research focusing on oncogene drivers has been conducted to investigate the regulatory mechanisms underlying the progression of LUAD. Targeted therapies have also been actively pursued. However, the complex immune microenvironment mechanism of LUAD remains poorly understood and requires further clarification. 7 NEDD1 has been suggested to exhibit upregulation in various cancer types and has the potential to serve as a diagnostic factor. To investigate its role in LUAD progression, a multivariate Cox model was constructed using clinical data from the Cancer Genome Atlas (TCGA). This model aimed to explore the relationship between NEDD1 expression, clinical features, and the promotion of LUAD progression. Additionally, the reliability of NEDD1 as a diagnostic marker for LUAD was assessed using ROC curves and survival assays.

To further explore the additional cancer-promoting effects of NEDD1, we performed differential analysis, PPI network interaction and functional enrichment analysis according to the expression of NEDD1. A NEDD1 knockdown experiment was designed to verify its effect on cell behavior. In addition, the distribution and expression of NEDD1 in single-cell data also led us to discuss its immune microenvironment and drug resistance with tumors.

## Materials and Methods

### Data acquisition

LUAD RNAseq and clinical data were downloaded from TCGA database, including 543 LUAD tissue and 59 normal tissue samples. GSE30219, including 85 LUAD tissue and 14 normal tissue samples, which as a validation dataset was obtained from GEO database. For the comparability of clinical features, 494 LUAD samples with relatively complete clinical information were screened from 543 LUAD samples of TCGA, including age, sex, TNM stage, survival status and survival time. The RNAseq data had been converted by log2(x+1). GSE198291 was used for the single-cell analysis, which downloaded from GEO database.

### Clinical samples acquisition and storage

The Cancer and adjacent tissues of 24 patients with minimally invasive lung adenocarcinoma, who had not undergone preoperative chemoradiotherapy, were collected from the First Affiliated Hospital of Guangxi Medical University. During the operation, the tumor mass and 5cm of tissue adjacent to the tumor were taken. After identification by the pathology department, LUAD tissue and adjacent tissue were left and stored in the refrigerator at -80℃. The clinical samples information and NEDD1 expression are shown in **Table S1**.

### Data mining, PPI, and functional enrichment analysis

We identified the expression of NEDD1 in pan-carcinoma. LUAD tissue samples from TCGA database, along with validation datasets GSE30219 were used to compare with NEDD1 expression at normal tissue samples (Tumor vs Normal). Additionally, NEDD1 was evaluated as a diagnostic and prognostic biomarker for LUAD by constructing receiver operating characteristic (ROC) curves and Kaplan-Meier (K-M) curves. Cox proportional hazard regression analysis was employed to examine the correlation between overall survival (OS) and NEDD1, age, gender, and TNM stages. Use R to visualize the results. Subsequently, GEPIA database and our clinical samples were used to further verify the expression of NEDD1 in different grades, and UALCAN database was used to compare the differences in the expression of NEED1 protein. 8, 9 The expression of NEDD1 was divided into high and low clusters by median, and the differentially expressed genes (DEGs) in LUAD were analyzed by DESeq2 package, and the protein interaction network was drawn from the genes with upregulated expression. 10 Finally, functional enrichment of DEGs was identified using GSEA and KEGG pathway enrichment assays. 11

### Cell culture

LUAD cell lines A549, 1299, and bronchial epithelial cell Beas-2b were obtained from the Chinese Academy of Sciences (Shanghai). A549 and Beas-2b cells were cultured in T25 culture flasks with DMEM medium (Gibco, USA) containing 10% FBS (Gibco) and 1% penicillin and streptomycin (Beyotime, China). H1299 was cultured in RPMI-1640 medium (Gibco). The cells were cultured with 5%CO2 and a constant temperature of 37℃.

### Cell transfection

We seed the cells into 6-well plates and incubate for 20 min at room temperature at the ratio recommended in the instructions using lipo8000 (Beyotime) with si-NEDD1, Opti-MEM (Gibco), and then transfect into the cells. All plasmids used in this study were prepared by Gensis Biotechnology Ltd (China). The corresponding siRNA sequences were as follows, siNEDD1-1: 5′-CACCAUUGACUUCCAUUCAAA-3′, siNEDD1-2: 5′-ACUAAGCAACUGGACUUUA-3′, and siNEDD1-3: 5′-UAGUGAGAAAGCAAGAUGUA-3′.

### RT-qPCR

The RNA was reverse transcribed from Fresh tissue samples or cells to cDNA (Prime Script RT Master Mix, Takara), and 10ug of the template was used for real-time quantitative PCR using 2XQ3 SYBR qPCR Master mix (ToloBio, China) and primers in the LightCycler480 system. Expression was calculated by 2-ΔΔCt and GAPDH as an internal reference. 12 The NEDD1 primer sequences were forward, 5′-AGTTGGTCACATCTGGTGCT-3′ and reverse, 5′-AGTGAACTGGCAACTCCAGC-3′.

**CCK8 Assay**

LUAD cells were adjusted to 1×10^4^/ml, and then 100ul cells were seeded into 96-well plates. After transfection, the cells were added with a 1:10 mixture of CCK8 and complete medium, and then cultured in an incubator for 4 hours. We measured the 450nm absorbance (450nm OD) at 0, 24, 48, and 72 hours.

### EdU assay

Forty-eight hours after transfection in 6-well plates, the culture medium was replaced with EdU solution (Beyotime) and incubated in an incubator for 2 hours. Fixed for 15 min with 4% paraformaldehyde solution, washed 3 times with PBS solution containing 3% BSA, then incubated with permeabilization solution at room temperature for 15 min, and finally reacted with light-protected reaction solution for 30 min. After incubation, the nuclei were stained and imaged under a fluorescence microscope.

### Transwell invasion and migration assays

We coated Matrigel Matrix (BD Bioscience, USA) into transwell chambers and incubated them for 2 hours. The transfected cells were resuspended in serum-free medium and adjusted to 2×10^5^/mL, and 750uL of complete medium was added outside the chamber. After 100uL of serum-free medium was added to the chamber, 200uL of cells were added and incubated in the incubator for 24h. Then, we fixed with paraformaldehyde and stained with crystal violet solution for 15min.

### Wound healing assay

We seeded A549 and H1299 cells into 6-well plates and transfected them, gently stroked the cells with a 200uL gun tip, washed them three times with PBS, photographed them under a microscope, then starved them with serum-free medium, and photographed the same area 24 hours later.

### Cell lysate preparation

To extract proteins, cells were lysed using RIPA lysis buffer (Beyotime) containing 1% PMSF (Beyotime). The protein concentration was assessed, and the samples were supplemented with buffer and boiled for 10 minutes prior to storage at -80 °C.

### WB assay

The protein samples, at a concentration of 20 μg per lane, were loaded onto an 8% SDS-PAGE gel along with pre-stained protein markers (WJ103, EpiZyme, China). The proteins were then transferred to PVDF membranes. Subsequently, the PVDF membranes were blocked using 5% skim milk for 1 h and washed three times with TBST. Next, the membranes were incubated overnight with primary antibodies at 4 °C. The next day, the membranes were incubated with goat anti-rabbit IgG and goat anti-mouse IgG secondary antibodies for 1 hour at room temperature. The following primary antibodies were used, E-Cadherin (Cat No. 20874-1-AP, 1:1000, Proteintech, China), N-Cadherin (Cat No. 22018-1-AP, 1:1000, Proteintech), Vimentin (Cat No. abs171412, 1:1000, absin, China), and NEDD1 (Cat No. 30637; 1:1000; Signalway Antibody, China). Normalization was performed with β-Actin (Cat No. 81115-1-RR, 1:5000, Proteintech).

### Single-cell analysis

Identify individual cell clusters in LUAD after performing quality control procedures and achieving dimensionality reduction and clustering by unified manifold approximation and projection (UMAP) using the Seurat package. 13 The clusters were manually annotated using marker genes through CellMarker database. 14 Finally, NEDD1 expression in different cell clusters was compared.

### Tumor immune cell infiltration

The GSVA package was utilized to conduct the single-sample gene set enrichment analysis (ssGSEA) on 24 distinct types of tumor-infiltrating immune cells (TIICs). 15

### Analysis of tumor mutation burden (TMB) and drug resistance

The maftools package was employed to generate oncoplots for the different NEDD1 clusters, with a cutoff value of 50%. Subsequently, a comparison was made between the tumor mutation burden (TMB) and immune checkpoint expressions in different NEDD1 clusters in LUAD. 16 Another package, oncoPredict, was utilized for the comparative analysis of resistance to chemotherapy and targeted therapy within distinct NEDD1 clusters. 17 The TIMER2.0 database was used to analyze the relationship between NEDD1 and different target genes of CAR-T therapies.

### Statistical analysis

All experiments conducted in this study were replicated three times to obtain average measurements. The obtained data were then analyzed and processed using SPSS 27.0 and GraphPad Prism 7.0 for statistical analysis and visualization. Additionally, ImageJ was employed for mathematical calculations based on the observed changes in the images. The statistical tests applied in this study included the t-test and one-way ANOVA to assess the significance of the results. The Wilcoxon rank sum test was utilized to determine the differences in clinical information. Significance levels were defined as p < 0.05, with the notation ns (not significant) for p > 0.05, * for p < 0.05, ** for p < 0.01, and *** for p < 0.001.

## Results

### NEDD1 serves as a credible prognostic marker in LUAD

**Figure 1:**
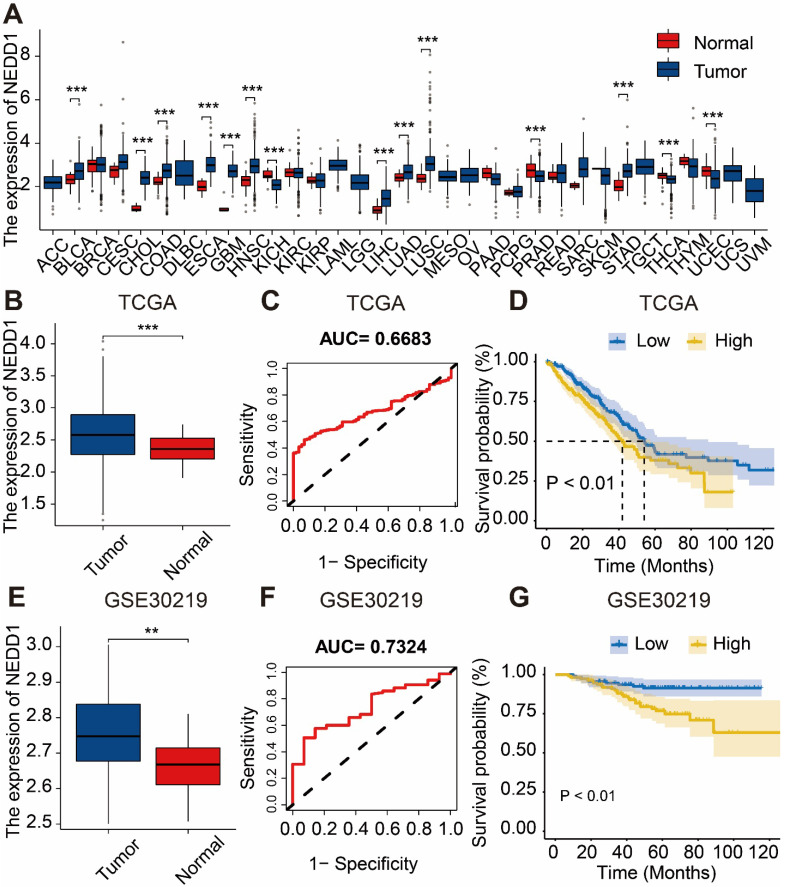
** Diagnostic value and survival predictive ability of NEDD1 for LUAD. (A)** Pan-cancer analysis of NEDD1 based on TCGA database. **(B)** Analysis of NEDD1 expression in LUAD and adjacent tissues based on TCGA database. **(C)** ROC curve analysis in the TCGA database. **(D)** K-M curve analysis in the TCGA database. **(E)** Analysis of NEDD1 expression in LUAD and adjacent tissues based on GSE30219. **(F)** ROC curve in the GSE30219. **(G)** K-M curve analysis in the GSE30219.

Based on TCGA database, we found that NEDD1 was highly expressed in various cancer types such as cervical cancer, lung adenocarcinoma, lung squamous cell carcinoma, etc. (**Figure 1A**). Analysis of the expression of NEDD1 and ROC curve results from the TCGA database revealed that NEDD1 exhibited upregulation in LUAD and displayed diagnostic value (AUC=0.6683). Additionally, K-M curve showed that the survival probability of patients with low expression of NEDD1 was significantly higher compared to those with high expression (P < 0.01) (**Figure 1B-D**). Similar results were obtained from the GSE30219 validation dataset (**Figure 1E-G**).

### Correlation between prognostic factors and NEDD1 expression

**Figure 2:**
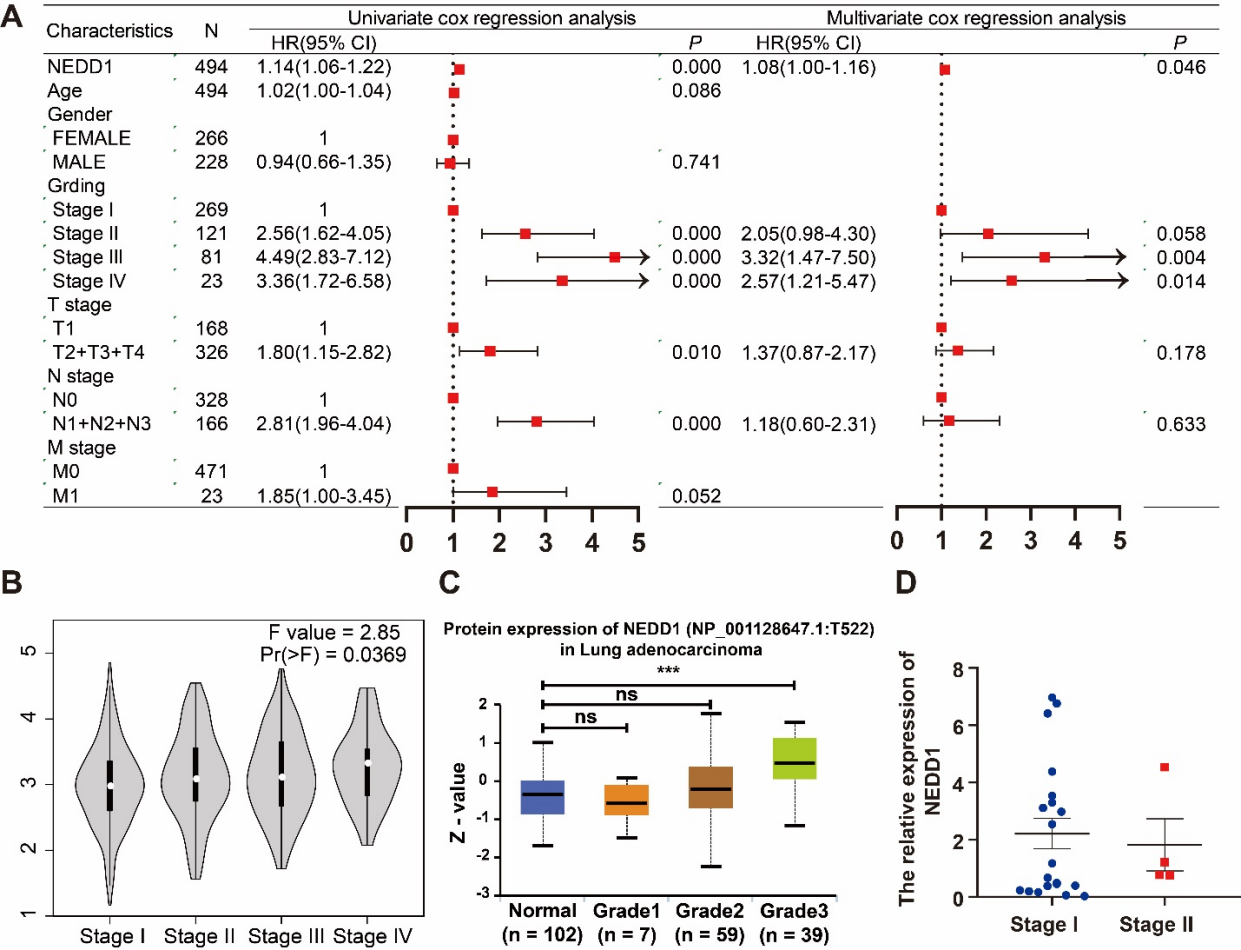
** NEDD1 and grades synergistically promoted LUAD progression. (A)** Univariate COX analysis of clinical features and NEDD1 in LUAD and multivariate COX analysis of independent prognostic factors in LUAD. **(B)** Comparison of NEDD1 expression in stage I, II, III, and IV. **(C)** The expression of NEDD1 protein was significantly increased in high grade (stage IV) compared with low grade (stage I). **(D)** There was no significant difference in NEDD1 expression of low grades LUAD in our samples.

Univariate COX analysis showed that NEDD1 (HR = 1.14 (1.06-1.22), P < 0.001), grades (stage I vs stage II, HR = 2.56 (1.62-4.05), p < 0.001; stage I vs stage III, HR = 4.49 (2.83-7.12), p < 0.001; and stage I vs stage IV, HR = 3.36 (1.72-6.58), p < 0.001), T-stage (T1 vs. T2+T3+T4, HR = 1.8 (1.15-2.82), P = 0.01), N-stage (N0 vs. N1+N2+N3, HR = 2.81 (1.96-4.04), P < 0.001), and M-stage (M0 vs. M1, HR = 1.85 (1-3.45), P = 0.052) as independent prognostic factors for LUAD. All independent prognostic factors were included in the multivariate COX analysis, and NEDD1 (HR = 1.08 (1-1.16), P = 0.046) and grades (stage I vs stage II, HR = 2.05 (0.98-4.3), p = 0.058; stage I vs stage III, HR = 3.32 (1.47-7.5), p = 0.004; and stage I vs stage IV, HR = 2.57 (1.21-5.47), p < 0.001) synergistically promoted LUAD progression (**Figure 2A**). According to the GEPIA database, the expression of NEDD1 in stage II, III, and IV of LUAD was significantly higher than that in stage I (**Figure 2B**). Furthermore, the results of UALCAN database showed that NEDD1 protein expression was not significantly different in low grades (stage I, II, and III), while NEDD1 protein expression was significantly increased in high grade (stage IV) (**Figure 2C**). The clinical samples we obtained showed no significant difference in NEDD1 expression of low grades LUAD (stage I vs stage II) (**Figure 2D**).

### Functional enrichment analysis of NEDD1

**Figure 3:**
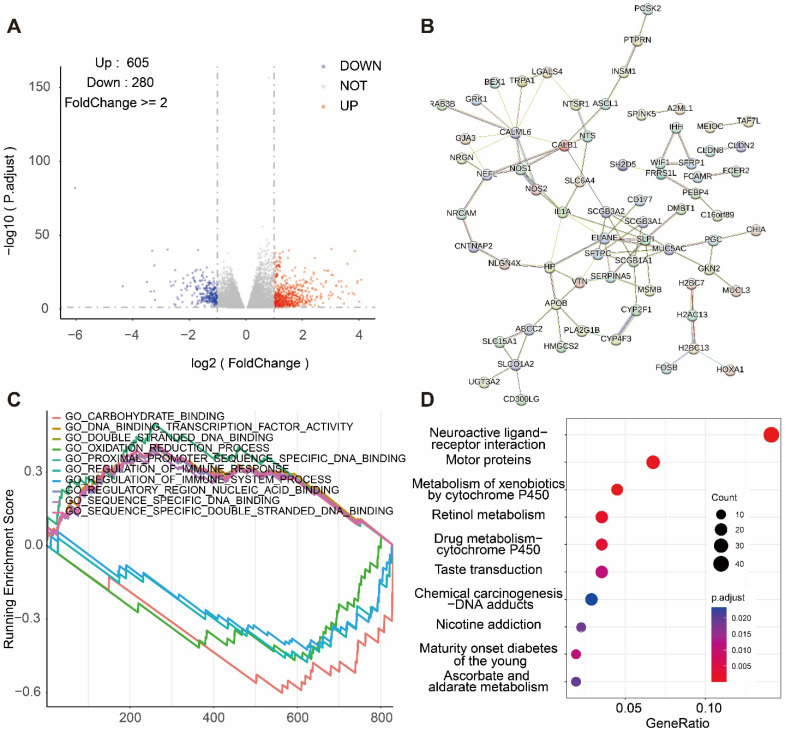
** Pathway analysis regarding NEDD1. (A)** DEGs associated with NEDD1 expression. **(B)** PPI network positively associated with NEDD1. **(C)** Analysis of the GSEA pathway associated with NEDD1. **(D)** KEGG enrichment analysis.

We selected 605 genes positively correlated with NEDD1 expression and 208 genes negatively correlated with NEDD1 expression from TCGA database (FoldChange ≥ 2, P < 0.05) (**Figure 3A**). The genes positively correlated with NEDD1 expression were mapped into PPI network (**Figure 3B**). Signaling pathways positively associated with NEDD1 include GO_PROXIMAL_PROMOTER_SEQUENCE_SPECIFIC_DNA_BINDING, GO_DNA_ BINDING_ _TRANSCRIPTION_ FACTOR_ ACTIVITY, GO_ DOUBLE_ STRANDED _DNA_ BINDING, GO_ SEQUENCE_ SPECIFIC_DNA BINDING, GO_ REGULATORY_ REGION_NUCLEIC_ ACID_BINDING, negatively related to the NEDD1 pathways including GO_CARBOHYDRATE_ BINDING, GO_ REGULATION OF _IMMUNE _SYSTEM_ PROCESS, GO_OXIDATION_ REDUCTION _PROCESS, GO_ REGULATION OF_ IMMUNE_ RESPONSE (GO = Gene ontology, **Figure 3C**). GSEA showed that NEDD1 expression was related to mitosis, chromosome segregation, cell cycle, etc., and KEGG enrichment analysis of NEDD1-related genes found that these genes were mainly related to neuroactive ligand-receptor interaction, motor proteins, metabolism of xenobiotics by cytochrome P450, retinol metabolism, drug metabolism--cytochrome P450, taste transduction, chemical carcinogenesis-DNA adducts, nicotine addiction ,maturity onset diabetes of the young, ascorbate and aldarate metabolism (P < 0.05, **Figure 3D**).

### NEDD1 expression in LUAD and knockdown in lung cancer cells

**Figure 4:**
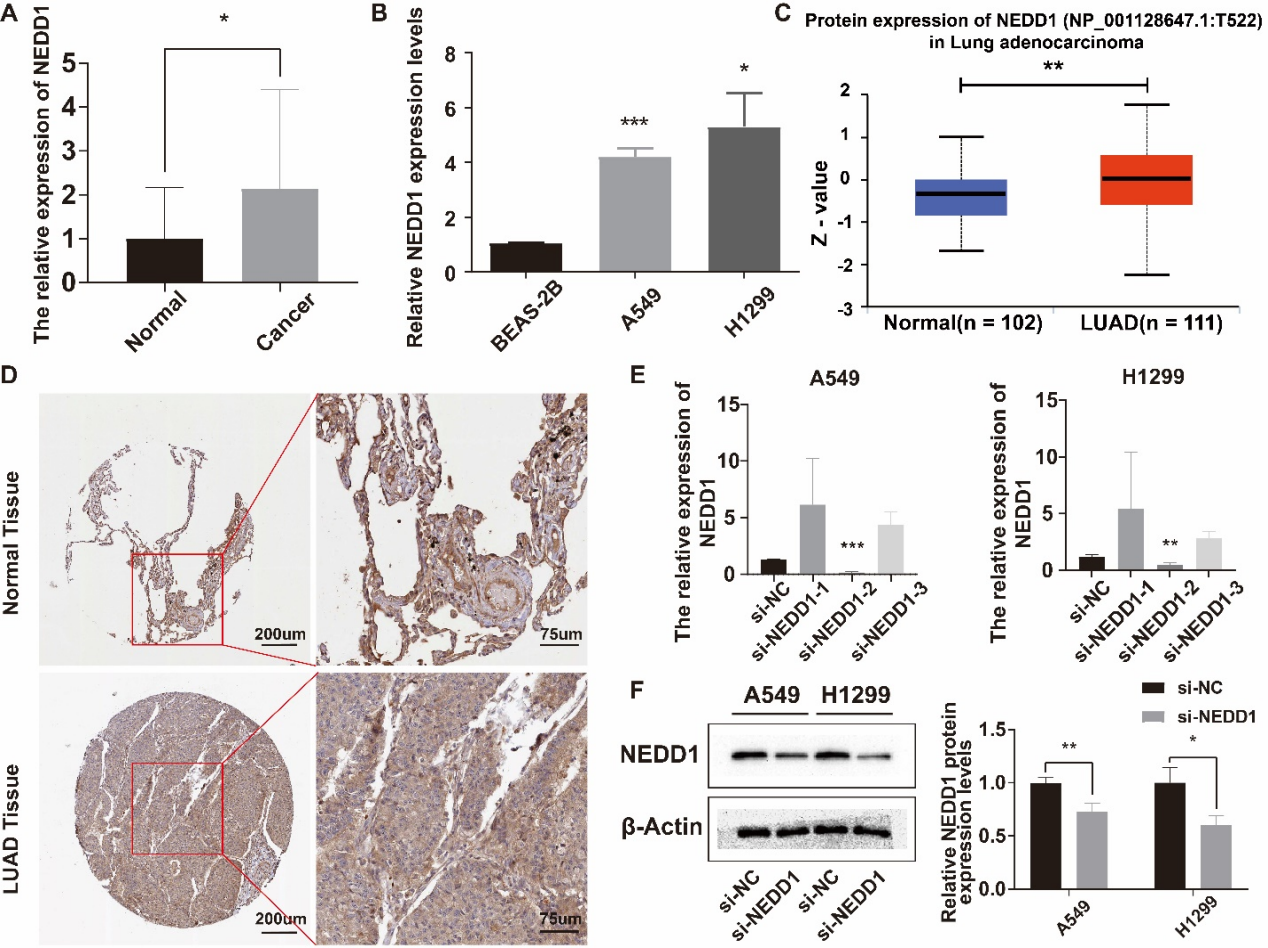
** Relative expression of NEDD1 in LUAD and lung cancer cells. (A)** Relative expression of NEDD1 in LUAD. **(B)** Relative expression of NEDD1 in lung cells. **(C)** The expression of NEDD1 protein in LUAD was significantly higher than that in normal tissues. **(D)** Immunohistochemistry of NEDD1 protein in cancer and para-cancerous based on HPA database. **(E)** Knockdown of NEDD1 in A549 and H1299 using different siRNAs. **(F)** NEDD1 protein expression in A549 and H1299 after NEDD1 knockdown.

The relative expression level of NEDD1 in LUAD tissues was higher than that in adjacent tissues (**Figure 4A**), and NEDD1 was highly expressed in A549 and H1299 (**Figure 4B**). UALCAN database showed that NEDD1 protein expression in LUAD was significantly higher than that in normal tissues (**Figure 4C**). Immunohistochemistry of NEDD1 protein in para-cancerous tissues and LUAD tissues (**Figure 4D**). After knocking down NEDD1 in A549 and H1299, the results showed that only si-NEDD1-2 had knockdown effect (**Figure 4E**). WB experiments showed that the protein expression of NEDD1 knockdown was significantly lower than that of the negative control group (**Figure 4F**).

### Knockdown of NEDD1 inhibited the proliferation of lung cancer cells

**Figure 5:**
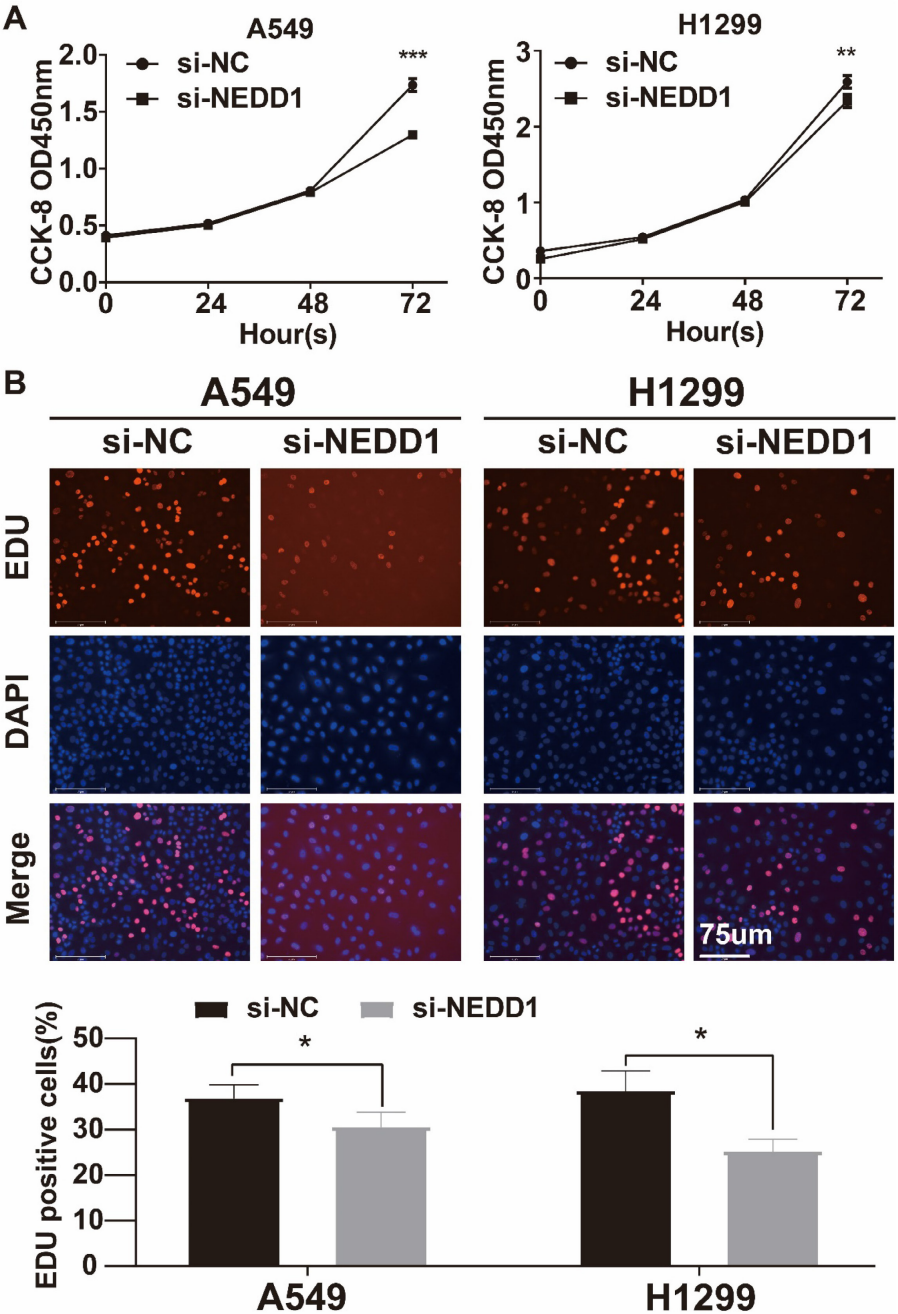
** The proliferation of lung cancer cells with NEDD1 knockout was inhibited. (A)** CCK8 indicated that cell proliferation was inhibited. **(B)** EdU assay was used to determine the proliferative capacity of NEDD1 silenced cells.

The CCK-8 assay revealed a significant decrease in cell absorbance over time in the silencing NEDD1 group, with a maximum decrease observed at 72 hours. This suggests that the inhibition of NEDD1 resulted in suppressed cell proliferation (**Figure 5A**). The EdU assay confirmed that the rate of EdU positive cells was significantly lower in the transfected group compared to the negative control group (**Figure 5B**).

### Knockdown of NEDD1 inhibited the migration, invasion, and EMT of lung cancer cells

**Figure 6:**
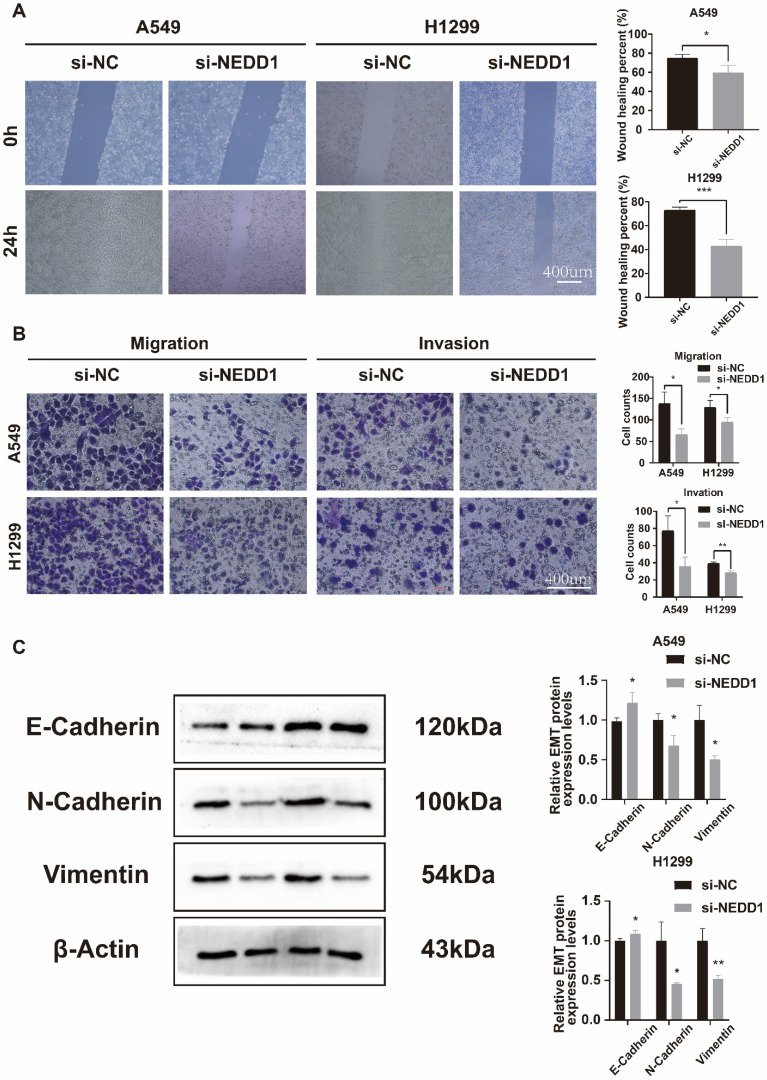
** After NEDD1 knockdown, the invasion, migration, and EMT processes of lung cancer cells were inhibited. (A)** Transwell assay assessed NEDD1 silenced cells invasion and migration ability. **(B)** Wound healing assay demonstrated cell migration ability. **(C)** The EMT of NEDD1 silenced cells was inhibited.

The Transwell assay demonstrated a significant reduction in the number of invaded and migrated A549 and H1299 cells following NEDD1 knockdown (**Figure 6A**). The wound healing assay was conducted to evaluate the migration ability of the cells. The results confirmed that the cells exhibited reduced migration ability after transfection with si-NEDD1 (**Figure 6B**). In Western blot (WB) experiments, β-actin was utilized as an internal reference. Following NEDD1 knockdown, the levels of Vimentin and N-cadherin were observed to decrease, whereas E-cadherin, indicative of the epithelial state, showed an increase (**Figure 6C**).

### Expression of NEDD1 in different cell types of LUAD

**Figure 7:**
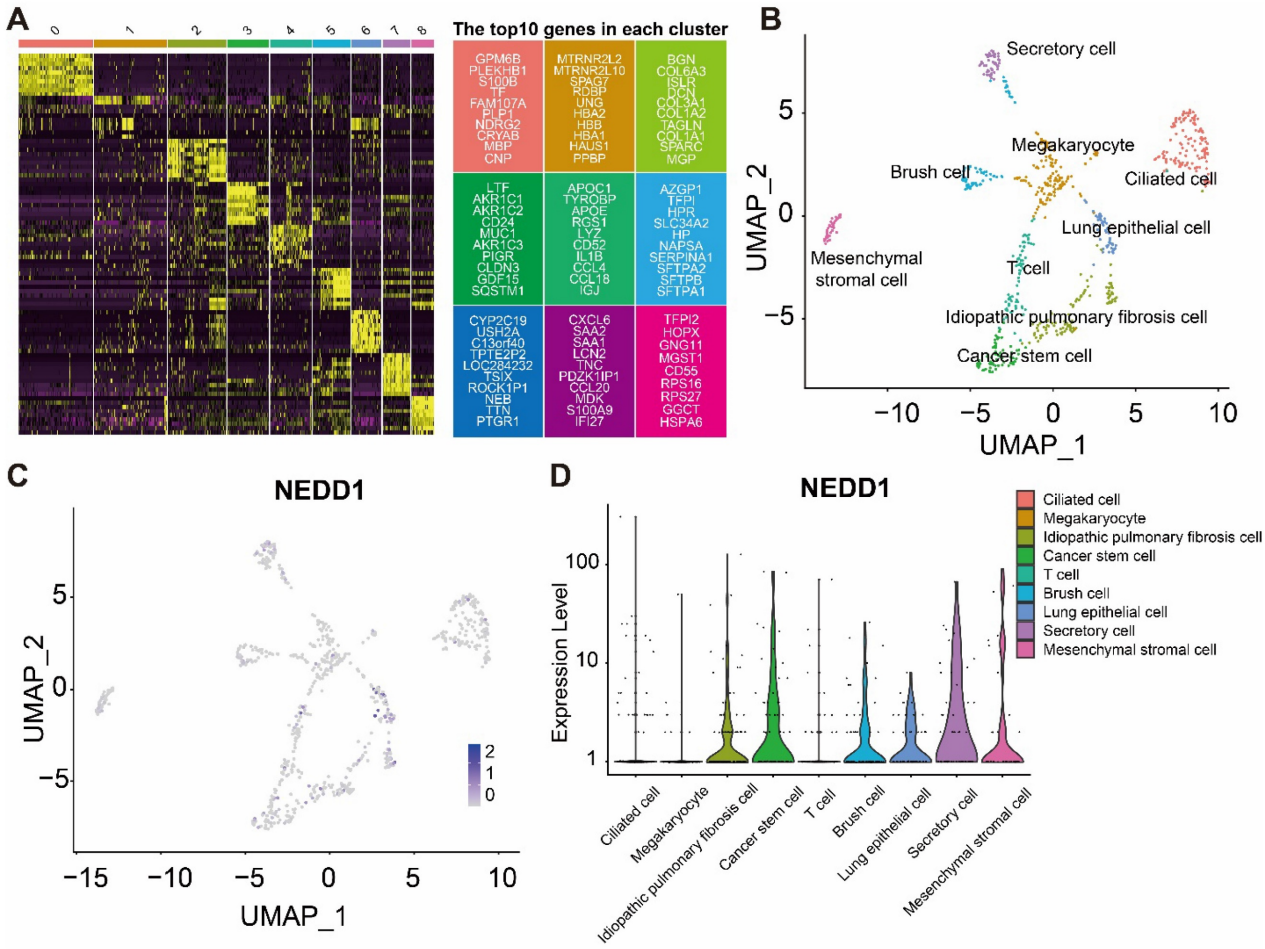
** Single-cell analysis shows the distribution of NEDD1 in LUAD. (A)** Expression profiles of marker genes in each cell cluster. **(B)** Distribution of NEDD1 in the UMAP plot of cells after dimensionality reduction clustering. **(C)** Nine cell types were labeled in UMAP. **(D)** NEDD1 expression in different cell types.

The heatmap shows the marker genes in different cell types (**Figure 7A**). Distribution of NEDD1 in different cell types (**Figure 7B**). UMAP map showed that nine types of cells were labeled, including chylous stromal cells, cancer stem cells, secretory cells, megakaryocytes, ciliated cells, idiopathic pulmonary fibrosis cells, lung epithelial cells, brush cells, and T cells (**Figure 7C**). NEDD1 is mainly expressed in idiopathic pulmonary fibrosis cells, cancer stem cells, brush cells, lung epithelial cells, secretory cells, and mesenchymal stromal cells (**Figure 7D**).

### NEDD1 plays a role in immune escape of LUAD

**Figure 8:**
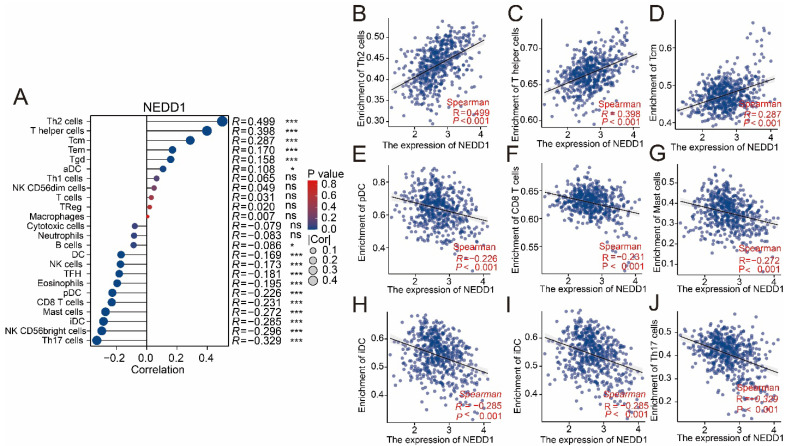
** Association of NEDD1 with immune cells. (A)** The ssGSEA analysis demonstrated correlation between NEDD1 expression and major immune cells. **(B-J)** Correlation between Th2 cells, T helper cells, Tcm, pDC, CD8+ T cells mast cells, iDC, CD56bright NK cells, Th17 cells and NEDD1.

The ssGSEA analysis showed that the positive correlation with NEDD1 expression was mainly immunosuppressive cells, while the main anti-tumor immune cells were negatively correlated (**Figure 8A**). The effect of NEDD1 expression in LUAD on immune cell infiltration showed that T helper 2 cells (Th2 cells, R = 0.449, P < 0.001), T helper cells (R = 0.398, P < 0.001), and central memory T cells (Tcm, R = 0.287, P < 0.001) were positively correlated with NEDD1 expression (**Figures 8B-D**). Furthermore, there is a strong negative correlation between the expression of NEDD1 with plasmacytoid dendritic cells (pDC, R = -0.226, P < 0.001), CD8+ T cells (R = -0.231, P < 0.001), mast cells (R = -0.272, P < 0.001), interdigitating dendritic cells (iDC, R = -0.285,P < 0.001), CD56bright NK cells (R = -0.296,P < 0.001), and T helper 17 cells (Th17 cells, R = -0.329,P < 0.001) (**Figure 8E-J**).

### The relationship between NEDD1 expression and drug resistance in LUAD

**Figure 9:**
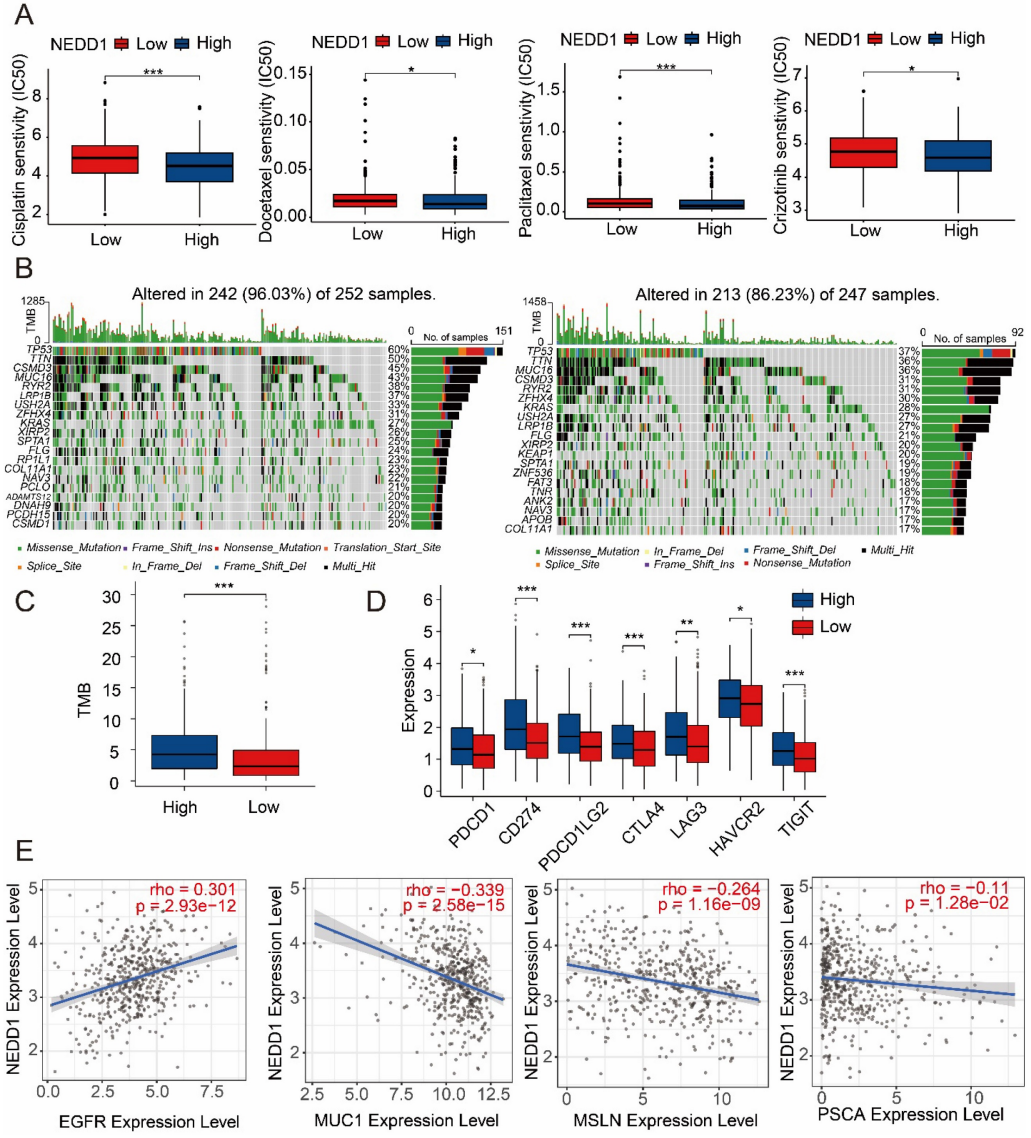
** NEDD1 increases drugs resistance to LUAD. (A)** Effect of NEDD1 expression on the IC50 of first-line therapeutic drugs. **(B)** Gene mutation profiles between different NEDD1 clusters. **(C)** The TMB of high NEDD1 clusters is significantly higher than that of low NEDD1 clusters. **(D)** Seven immune checkpoints were expressed increased in high NEDD1 clusters. **(E)** The effect of NEDD1 on CAR-T therapy in LUAD.

NEDD1 expression affects the sensitivity of LUAD to first-line therapies. Therefore, we assessed resistance of NEDD1 expression to common first-line chemotherapy drugs and found that high NEDD1 clusters had lower semi-inhibitory concentrations (IC50) than low NEDD1 clusters in Cisplatin, Docetaxel, Paclitaxel, and Crizotinib (**Figure 9A**). TMB as a predictive biomarker for different ICI therapies can affect LUAD resistance, and **Figure 9B** shows the difference in gene mutations in the high and low clusters of NEDD1. The TMB of high NEDD1 clusters is significantly higher than that of low NEDD1 clusters (**Figure 9C**). Meanwhile, seven immune checkpoints (PDCD1 CD274, PDCD1LG2, CTLA4, LAG3, HAVCR2, TIGIT) were expressed increased in high NEDD1 clusters (**Figure 9D**). In addition, we explored the impact of NEDD1 on the treatment of CAR-T for lung cancer. The results showed that EGFR was positively correlated with NEDD1 expression and negatively correlated with MUC1, MSLN and PSCA, indicating that PD-L1-CAR-T cells may have hope for new therapeutic prospects for LUAD patients with high NEDD1 expression (**Figure 9E**).

## Discussion

Currently, the treatment strategy for lung cancer is based on the size of the primary tumor and the presence of distant metastasis. With the widespread use of low-dose CT, early-stage and low-invasive lung cancers are often treated with radical surgery, leading to a five-year survival rate of 70% for patients. 18, 19 Patients diagnosed with stage II and III lung cancer who undergo radical surgery following preoperative systemic drug therapy to reduce the size of the primary tumor have shown a three-year survival rate of 56%. Additionally, the utilization of targeted chemotherapy drugs and immune checkpoint inhibitors has further extended survival in these patients. 20-22 Nevertheless, the five-year survival rate for patients with advanced lung cancer remains less than 5%. The treatment strategy for advanced lung cancer is still undecided due to the diverse mutation sites of the disease and the absence of specific membrane surface antigens. 23, 24

Chromosomal instability (CIN) is believed to have a significant impact on the multistep process of tumorigenesis, particularly in tumor growth, initiation, and response to treatment. Abnormal expression of regulated chromosomal segregated cyclin and spindle-assembly checkpoint (SAC) can lead to improper separation of one or more chromosomes. 25-27 Hence, targeting microtubule (MT) dynamics to disrupt mitotic spindle assembly and reduce chromosomal separation and chromosomal instability (CIN) during mitosis in lung cancer cells has been widely employed as a therapeutic strategy using chemotherapy drugs in cancer treatment. 28 Previous research has demonstrated that during mitosis, Aurora A kinase facilitates microtubule (MT) nucleation around chromatin through the phosphorylation of NEDD1. This phosphorylation event promotes the proper assembly of a functional mitotic spindle. 29 Furthermore, a study by Manning JA et al. revealed that NEDD1 has a significant role in the senescence of mouse embryonic fibroblasts (MEFs). Depletion of NEDD1 was found to decrease the level of γ-tubulin in centrosomes, resulting in the fragmentation of MEFs centrosomes and subsequent premature senescence. 30 Based on the findings, there is a compelling rationale to consider NEDD1 as a potential novel therapeutic target for tumors.

Our study discovered that NEDD1 displays abnormal upregulation in various types of solid tumors, including breast, liver, colon, esophageal, and lung cancers. Consequently, we employed NEDD1 and clinical features to establish a prognostic model for LUAD, aiming to assess the prognostic value of NEDD1 as a marker specifically for LUAD. Moreover, the correlation between prognostic factors and NEDD1 expression was used to verify their synergistic contribution to LUAD progression. In addition to the previously identified role of NEDD1 in regulating cell mitosis and proliferation, our analysis utilizing the KEGG enrichment approach revealed potential associations between NEDD1 and neuroactive ligand-receptor interaction pathways, motor protein, P450, as well as immune response regulation. The chromosome 15q25.1 locus has been identified as the primary susceptibility region for lung cancer. The genes within this region, affected by single-nucleotide polymorphisms (SNPs) and haplotypes, have been found to contribute to the neuroactive ligand receptor interaction pathway and gated channel activity, as determined by KEGG enrichment analysis. 31 Consequently, the neuroactive ligand receptor interaction was identified as a susceptibility risk factor for the development of lung cancer. Studies have demonstrated the involvement of motor protein in carcinogenesis, wherein a specific set of motor proteins, namely KIF11, KIF15, and KIF4A, have been found to exhibit a correlation with NEDD1 expression. Besides, LUAD patients with elevated levels of these motor proteins have been shown to have poorer survival outcomes. 32 CIN often caused by environmental factors such as smoking or exposure to dangerous carcinogens, is thought to be the cause of many cancers, and cytochrome P450 is essential for the metabolism of carcinogens, but this effect can be interrupted by crosstalk by other transcription factors. 33, 34 In summary, the involvement of NEDD1 in the regulation of proliferation, migration, invasion, and epithelial-mesenchymal transition of lung cancer cells has been confirmed through NEDD1 knockdown experiments. However, the precise role of NEDD1 in the tumor microenvironment of lung adenocarcinoma (LUAD) remains unknown. Additionally, its potential contribution to tumor immune invasion and its impact on drug resistance are areas of particular interest that warrant further investigation.

Cancer stem cells are a class of cells that are rare in number, but highly malignant, and eventually develop into tumors through their powerful ability to regenerate, proliferate and differentiate. 35 With the continuous growth and accumulation of tumor cells, due to the high metabolism and oppression of tumor cells, local tissues appear hypoxia and necrosis, and release many inflammatory factors, thereby recruiting mesenchymal stem cells to perform scar repair on tissue damage sites and then differentiate into idiopathic pulmonary fibrosis cells and mesenchymal stromal cells. 36 Furthermore, as the tissue repair process progresses and replaces the inflammatory process, there is a gradual decrease in the inflammatory response. This reduction in inflammation facilitates the restoration of tissue homeostasis and enables the reorganization of normal cells, including brush cells, lung epithelial cells, and secretory cells. 37 The newly formed tumor microenvironment reduces exposure to tumor antigens and enables immune escape of tumor cells by recruiting immunosuppressive cells. 38 We analyzed the distribution of NEDD1 in lung cancer tissues using the single-cell dataset of LUAD in the GEO database, and the results were consistent with the growth and development pattern of tumor cells. At the same time, the expression of NEDD1 in LUAD increases, which increases the infiltration of a variety of immunosuppressive cells and decreases the infiltration of major anti-tumor immune cells.

Based on the functional characteristics of NEDD1 in LUAD and the effect on tumor-infiltrating immune cells, we further analyzed the relationship between NEDD1 expression and drug resistance in chemotherapy and targeted therapy drugs. Tumor cells grow and metabolize vigorously, and cisplatin induces tumor cell death by acting on purine and pyrimidine bases in DNA. 39 Paclitaxel, or docetaxel, which act on the microtubule paclitaxel site, cause mitosis arrest of cells by destroying the microtubule network, but it is also believed that paclitaxel exerts its anti-cancer effect by causing chromosomal malfusion on the multipolar spindle of cancer cells, increasing chromosomal instability. 28, 40 Cisplatin plus pemetrexed, paclitaxel, or docetaxel is the first-line treatment of choice in driver-negative cases of advanced LUAD. Crizotinib is recommended for patients with positive ALK and ROS1 fusion genes, but unfortunately our results did not show a correlation between NEDD1 and other driver genes. 41 Administering the mentioned drugs to LUAD patient's dependent on NEDD1 could potentially lead to improved long-term outcomes. Additionally, tumor cells with high tumor mutational burden (TMB), influenced by genetic mutations, may experience the exposure of new antigens. This exposure can activate immune cells, which in turn can target and eliminate tumor cells, ultimately achieving anti-tumor effects. 24 However, NEDD1 has shown unexpected effects by inhibiting immune cells through the upregulation of immune checkpoints on tumor cells, and more seriously, mutations in genes such as EGFR, ALK, KRAS, and STK11 can worsen the response to immune checkpoint inhibitors (ICIs). 42-44 In contrast, the efficacy of CAR-T cell therapy in lung cancer has been limited due to the absence of distinct surface antigens. Currently, CAR-T cell therapy has shown some advancements in targeting specific antigens such as PD-L1, EGFR, MUC1, MSLN, and PSCA in lung cancer treatment. 23, 45 However, NEDD1 has exhibited resistance to most CAR-T cell therapies. In conclusion, we posit that NEDD1-dependent LUAD lacks the requisite therapeutic targets for neoadjuvant chemotherapy, and the presence of tolerance to ICIs and CAR-T cell therapies further exacerbates LUAD resistance. Consequently, NEDD1 could serve as a promising new therapeutic target for LUAD, providing an emerging treatment avenue by directly inducing cancer cell death or increasing the sensitivity of LUAD to immune checkpoint inhibitors and CAR-T cell therapies.

## Conclusions

NEDD1 serves as a dependable prognostic marker for LUAD, as it elevates the risk of developing this type of lung cancer through synergistic interactions with various cancer susceptibility genes. Cytobehavioral experiments targeting NEDD1 through knockdown approaches have also confirmed its promotion of proliferation, migration, invasion, and epithelial-mesenchymal transition in lung cancer cells. Furthermore, NEDD1-mediated chromosomal instability can contribute to an increased risk of immune evasion and resistance to frontline chemotherapy drugs and immunotherapeutic agents in tumor cells. With these insights, NEDD1 holds significant promise as a novel therapeutic target for LUAD.

## Supplementary Material

Supplementary table.
